# Determination of strain relaxation in InGaN/GaN nanowalls from quantum confinement and exciton binding energy dependent photoluminescence peak

**DOI:** 10.1038/s41598-018-26725-6

**Published:** 2018-05-30

**Authors:** Sandeep Sankaranarayanan, Shonal Chouksey, Pratim Saha, Vikas Pendem, Ankit Udai, Tarni Aggarwal, Swaroop Ganguly, Dipankar Saha

**Affiliations:** Applied Quantum Mechanics Laboratory, Indian Institute of Technology Bombay, Powai, Mumbai, 400076 India

## Abstract

GaN based nanostructures are being increasingly used to improve the performance of various devices including light emitting diodes and lasers. It is important to determine the strain relaxation in these structures for device design and better prediction of device characteristics and performance. We have determined the strain relaxation in InGaN/GaN nanowalls from quantum confinement and exciton binding energy dependent photoluminescence peak. We have further determined the strain relaxation as a function of nanowall dimension. With a decrease in nanowall dimension, the lateral quantum confinement and exciton binding energy increase and the InGaN layer becomes partially strain relaxed which decreases the piezoelectric polarization field. The reduced polarization field decreases quantum confined Stark effect along the c-axis and increases electron-hole wave-function overlap which further increases the exciton binding energy. The strong dependency of the exciton binding energy on strain is used to determine the strain relaxation in these nanostructures. An analytical model based on fractional dimension for GaN/InGaN/GaN heterostructures along with self-consistent simulation of Schrodinger and Poisson equations are used to theoretically correlate them. The larger effective mass of GaN along with smaller perturbation allows the fractional dimensional model to accurately describe our system without requiring first principle calculations.

## Introduction

GaN based nanostructures are being heavily explored for light emitting sources where quantum confinement and larger surface to volume ratio provide many advantages^1–7^. Various InGaN/GaN and AlGaN/GaN based quantum-wells, walls, wires and dots are used as efficient light sources for infrared to ultra-violet emission demonstrating higher quantum efficiency, photo- and electro-luminescence, large exciton binding energy, lower threshold current for lasers, and lower efficiency droop^8–16^. The emission wavelength is among the most important parameters which characterizes these sources. The emission wavelength is primarily determined by quantum confinement and resulting exciton binding energy. The quantum confinement in these structures is asymmetric in nature due to polarizations leading to other effects including quantum confined Stark effect (QCSE)^17–19^. In addition, due to the triangular nature of the quantum well at lower energy, the barrier can dynamically evolve and the well region can itself act as its own barrier. The strain relaxation in these nanostructures is significant which sometime leads to a wide variety of reports on emission wavelengths due to a change in polarization^20–26^. In addition, a discrepancy between experiments and theory may creep in due to an incorrect estimation or complete ignorance of the strain relaxation. Hence, it is imperative to determine strain relaxation in GaN based nanostructures for better understanding and accurate design of the light emitting sources.

In this work, we have bridged the above-mentioned gap where we have shown that peak emission wavelength can be considered as a signature of the strain relaxation in GaN nanostructures by considering accurate nature of quantum confinement through fractional dimension. The photoluminescence measurements are done at low power to avoid perturbing the system away from the equilibrium. We have used GaN nanowalls as the host device^27,28^. Nanowalls of different dimensions and degree of strain relaxations are considered. The strain relaxation changes the polarization of GaN nanostructures, which in turn changes the electrostatic confinement potential and exciton binding energy. Hence, the change in the peak emission wavelength which is determined by the bound states in a quantum well and exciton binding energy is an indicator for the strain relaxation when the exciton binding energy is accurately modeled by considering fractional dimension^29,30^. The strain is found to relax up to 70% for 10 nm nanowall.

The analytical method based on fractional dimensional space is earlier reported to accurately determine the exciton binding energy in semiconductor quantum wells. We have extended the model for nanowalls having an asymmetric potential well. The excitons in an anisotropic solid, like a quantum well or a quantum wire, are treated as the ones in an isotropic solid with a fractional dimension (α), wherein the degree of anisotropy determines the dimension. The varied electron-hole interaction with dimension is the source of anisotropy in these nanostructures. A knowledge of the fractional dimension α in the model can be used to estimate the exciton binding energies continuously from three-dimensional (3D) to one-dimensional (1D) structures.

**Figure 1:**
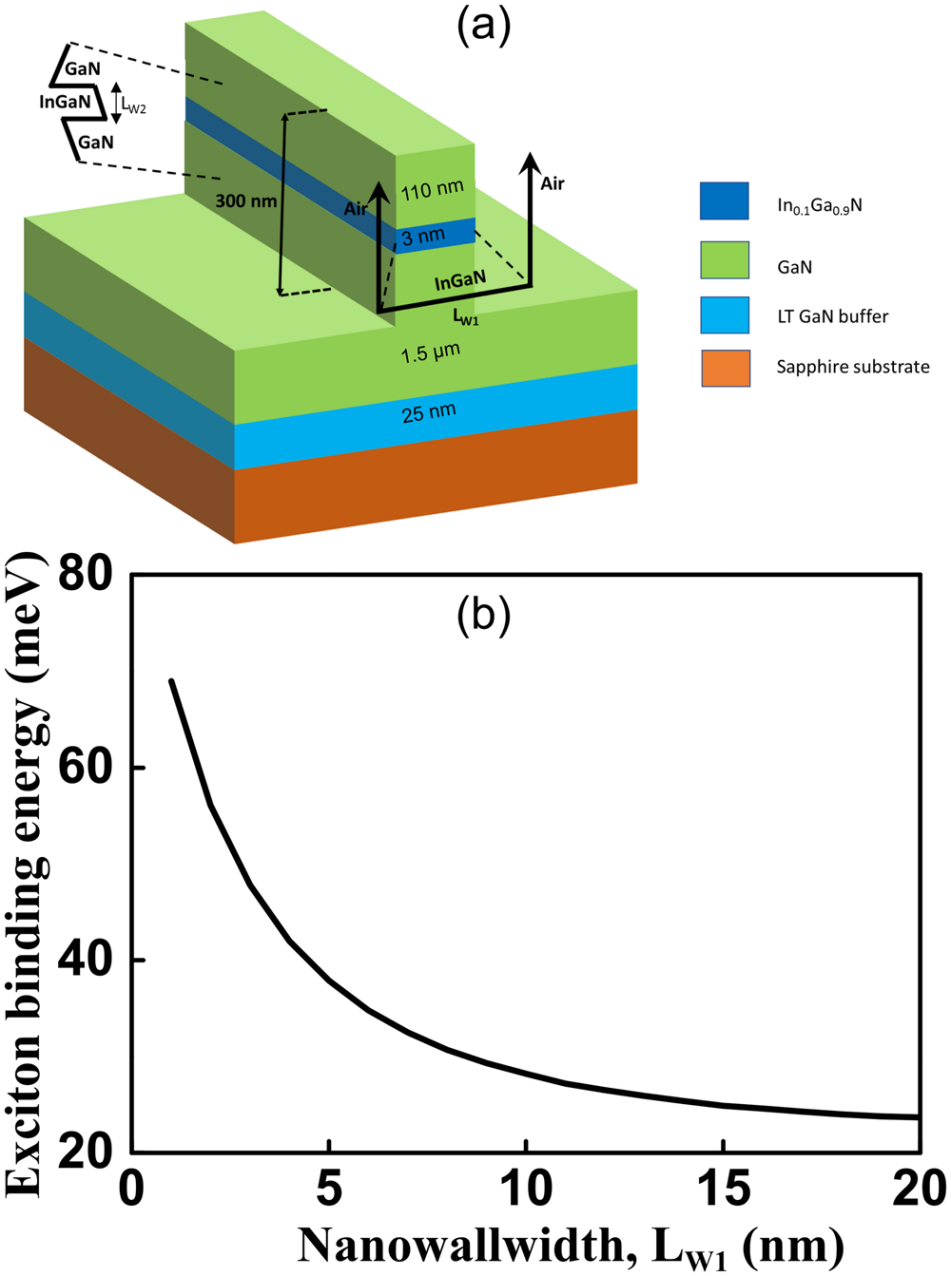
(**a**) A schematic of the heterostructure and the confining potential for both the directions are shown; (**b**) plot of the exciton binding energy (E_*b1*_) as a function of the nanowall width (L_*w1*_).

The relative motion of a free exciton in a quantum structure of fractional dimension α can be described using the Schrodinger equation as^30^:

1 $$[\frac{-{\hslash }^{2}}{2\mu {r}^{\alpha -1}}\frac{\partial }{\partial r}({r}^{\alpha -1}\frac{\partial }{\partial r})+\frac{{l}^{2}}{2\mu {r}^{2}}-\frac{{e}^{2}}{4\pi {\epsilon }{{\epsilon }}_{0}r}]{\rm{\Psi }}(r,\,\theta )=(E-{E}_{g}){\rm{\Psi }}(r,\,\theta )$$

where µ is the electron (m_e_)-hole (m_h_) reduced effective mass given by $1/\mu =1/{m}_{e}+1/{m}_{h}$, ɛ is the dielectric constant, r is the electron-hole distance, E_g_ is the effective energy band-gap, and *l*^2^ is the angular momentum operator. The discrete bound-state energies (*E*_*n*_) and orbital radii (*a*_*n*_) are obtained from the solution as, ${E}_{n}={E}_{g}-{E}_{0}/{[n+(\alpha -3)/2]}^{2}$ and ${a}_{n}={a}_{0}{[n+(\alpha -3)/2]}^{2}$; where n is the principal quantum number, *E*_0_ and *a*_0_ are the effective Rydberg constant and effective Bohr radius given by, ${E}_{0}=({\varepsilon }_{0}/\varepsilon )(\mu /{\mu }_{0}){R}_{H}$ and ${a}_{0}=(\varepsilon /{\varepsilon }_{0})({\mu }_{0}/\mu ){a}_{H}$, respectively. Here, *R*_*H*_ and *a*_H_ are the Rydberg constant and Bohr radius, respectively, and µ_o_ is the free electron mass. Figure 1(a) shows a schematic of the nanowall and the confining potential for both the directions. The heterostructure is grown by metal-organic chemical vapor deposition (MOCVD) on a sapphire substrate. A low-temperature (LT) GaN buffer layer (25 nm) is first deposited before the growth of the active device heterostructure. The active region consists of a single quantum well of 3 nm In_0.1_Ga_0.9_N layer and GaN as the barrier layer. The entire heterostructure is unintentionally doped. The height of the nanowall is ~300 nm. The exciton binding energy (*E*_*b*_) is obtained as the difference between the effective energy bandgap (*E*_*g*_) of the material and the first bound state energy of the exciton (E_n = 1_) as, ${E}_{b}={E}_{g}-{E}_{1}$. Thus, the binding energy for 1 s exciton is given by ${E}_{b}={[2/(\alpha -1)]}^{2}{E}_{0}$. The α is related to a dimensionless pertinent parameter β which is the average electron-hole distance normalized with respect to the effective Bohr radius in the direction of quantum confinement as^29^:

2 $$\alpha =3-{e}^{-\beta }$$

We have extended the above model in the case of nanowalls and asymmetric confining potential for GaN based heterostructures. The two-dimensional (2D) confinement are in the orthogonal direction and perpendicular to the length of the nanowall. The InGaN active layer is confined (i) horizontally by an infinite potential well with air on both sides and (ii) vertically by the GaN barriers in a finite potential well which is asymmetric owing to the spontaneous and piezoelectric polarizations. The infinite potential well condition is analyzed by choosing the dimensionless pertinent parameter *β* = *L*_*W1*_*/2a*_0_, where *L*_*W*1_ is the effective nanowall width. Hence, the exciton binding energy due to the lateral confinement is given by, ${E}_{b1}={E}_{0}{[1-\frac{1}{2}{e}^{-{L}_{w1}/2{a}_{0}}]}^{-2}$. Figure 1(b) shows the increasing exciton binding energy due to increasing lateral confinement of the nanowalls.

**Figure 2:**
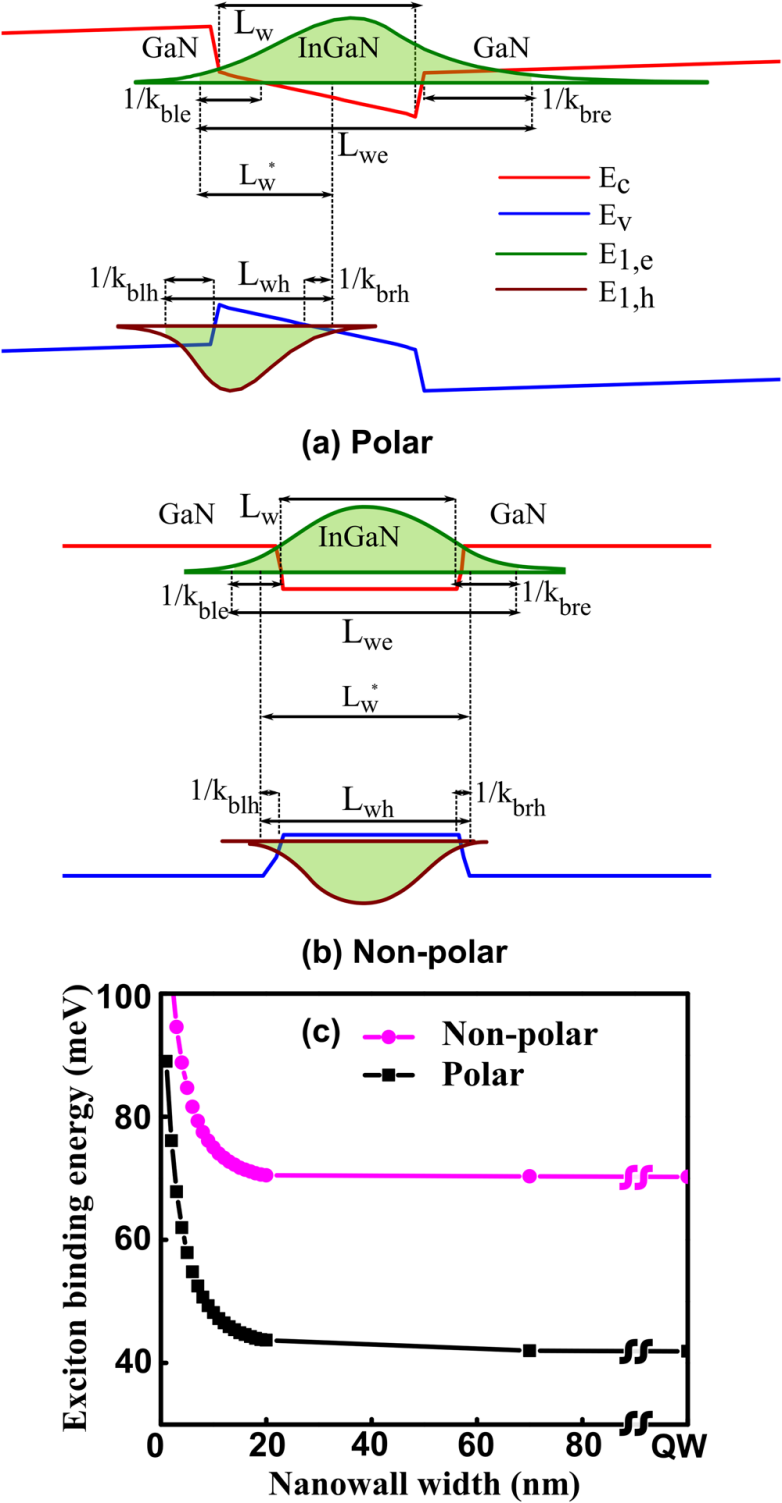
The finite (**a**) polar and (**b**) non-polar GaN/InGaN/GaN quantum well with electron and hole wave functions spreading into the barrier region are shown. Effective well width L_w_* evolves dynamically when the polarization field changes; (**c**) the width dependent exciton binding energies for the strained (polar) and relaxed (non-polar) InGaN/GaN nanowalls.

The confinement due to the InGaN/GaN finite potential well along the c-direction is asymmetric in nature and the potential profile is obtained by solving the Schrodinger and Poisson equations self-consistently and taking into account the effects of polarizations. It may be noted that the well width *L*_*W*2_ is fixed at 3 nm in this case along the c-direction. As it is a finite potential well, the electron and hole envelope functions are spread into the barriers. The bound-state energies of a particle confined in a rectangular potential well is given by the well-known transcendental equations and the characteristic wave vectors in well (*k*_*w*_) and barrier (*k*_*b*_) are given by, ${k}_{w}=\sqrt{2{m}_{w}{E}_{p}}/\hslash$ and ${k}_{b}=\sqrt{2{m}_{b}(V-{E}_{p})}/\hslash$, respectively, where *m*_*w*_ and *m*_*b*_ are the effective masses of the carriers in the well and the barrier, and *V* is the quantum well depth. Taking into account the spread of the envelope functions (shown in Fig. 2(a,b)) into the barriers, the effective well width, ${L}_{w(e,h)}$ for electron (hole) is defined as:

3 $${L}_{we(h)}=\frac{1}{{k}_{ble(h)}}+{L}_{w2}+\frac{1}{{k}_{bre(h)}}$$

where *l* and *r* in the suffix denote the right and left side of the quantum well, respectively, and ${L}_{w2}$ is the quantum well width as shown in Fig. 2(a,b). Due to the presence of the high internal electric field in the InGaN region, the quantum well is not perfectly rectangular and the confining potential width can be less than the physical thickness of the quantum well. The spatial extent of the exciton in the z direction is represented by a dynamic effective well width *L*_w_^*^ which is the distance over which electron and hole envelope functions can overlap as shown in Fig. 2(a).

The effective Bohr radius varies with the position-dependent effective masses due to the spread of the envelope function in the barrier. There can be two different scenarios depending on the position of the bound state with respect to the barrier: (1) GaN/InGaN/GaN structure with GaN forming the barriers and (2) InGaN/InGaN/GaN if the bound state is closer to the conduction band minima (see Fig. 2(a)) which causes the left barrier to be the InGaN layer itself. In order to account for this mismatch in the electron (hole) effective masses between the well (${m}_{we(h)}$) and barrier (${m}_{bl(r)e(h)}$) regions, three scaling parameters are defined as*, S*_*we(h)*_ = *L*_*W2*_*/L*_*we(h)*_, *S*_*ble(h)*_ = *[1/k*_*ble(h)*_*]/L*_*we(h)*_, and *S*_*bre(h)*_ = *[1/k*_*bre(h)*_*]/L*_*we(h)*_. The weighted effective mass (m*_e(h)_) and dielectric constant ${\varepsilon }^{\ast }$ are thus given by,

4 $${m}_{e(h)}^{\ast }={S}_{we(h)}{m}_{we(h)}+{S}_{ble(h)}{m}_{ble(h)}+{S}_{bre(h)}{m}_{bre(h)}$$

5 $${\varepsilon }^{\ast }=\sqrt{{S}_{we}{S}_{wh}}{\varepsilon }_{w}+\sqrt{{S}_{ble}{S}_{blh}}{\varepsilon }_{bl}+\sqrt{{S}_{bre}{S}_{brh}}{\varepsilon }_{br}$$

Now the effective Bohr radius (*a*_0_^*^) and Rydberg constant (*E*_0_^*^) are obtained as:$\,{a}_{0}^{\ast }=\frac{{\varepsilon }^{\ast }}{{\varepsilon }_{0}}\frac{{m}_{0}}{{\mu }^{\ast }}{a}_{H}$ and ${E}_{0}^{\ast }=\frac{{\varepsilon }_{0}}{{\varepsilon }^{\ast }}\frac{{\mu }^{\ast }}{{m}_{0}}{R}_{H}$, where ${\mu }^{\ast }$ is the reduced exciton effective mass given by 1/µ^*^=1 /m^*^_e_+1 /m^*^_h_. The dimensionless pertinent parameter in this case is, *β* = *L*_W2_^*^/2a_0_^*^. The exciton binding energy due to the vertical confinement is then obtained as, ${E}_{b2}={E}_{0}^{\ast }{[1-\frac{1}{2}{e}^{-{L}_{w2}^{\ast }/2{a}_{0}^{\ast }}]}^{-2}$.

It is interesting to note that the exciton binding energy for the InGaN quantum well is estimated to be 26 and 51 meV with and without polarization fields, respectively. This emphasizes the strong dependency of the exciton binding energy on polarizations which in turn is dependent on the strain. The larger exciton binding energy in the absence of polarizations is due to the reduced QCSE and a larger electron and hole wave-function overlap.

Having determined the exciton binding energy due to confinements in two orthogonal directions, the exciton binding energy for the nanowall is estimated as, *E*_*b*_ = *E*_*b1*_ + *E*_*b2*_. The nanowalls with very small width tend to relax the strain which can change the polarization. Hence, the strained and relaxed nanostructures provide the lower and upper bounds for the exciton binding energy, respectively. Figure 2(c) shows the width dependent exciton binding energies for the strained and relaxed InGaN/GaN nanowalls. The degree of strain relaxation changes with wall width. Hence, the actual exciton binding energy for a nanowall of fixed width will lie between the two limiting cases. It is now left to determine the strain relaxation for various nanowall widths from given exciton binding energies. For that purpose, nanowalls are fabricated and photoluminescence measurements are carried out.

**Figure 3:**
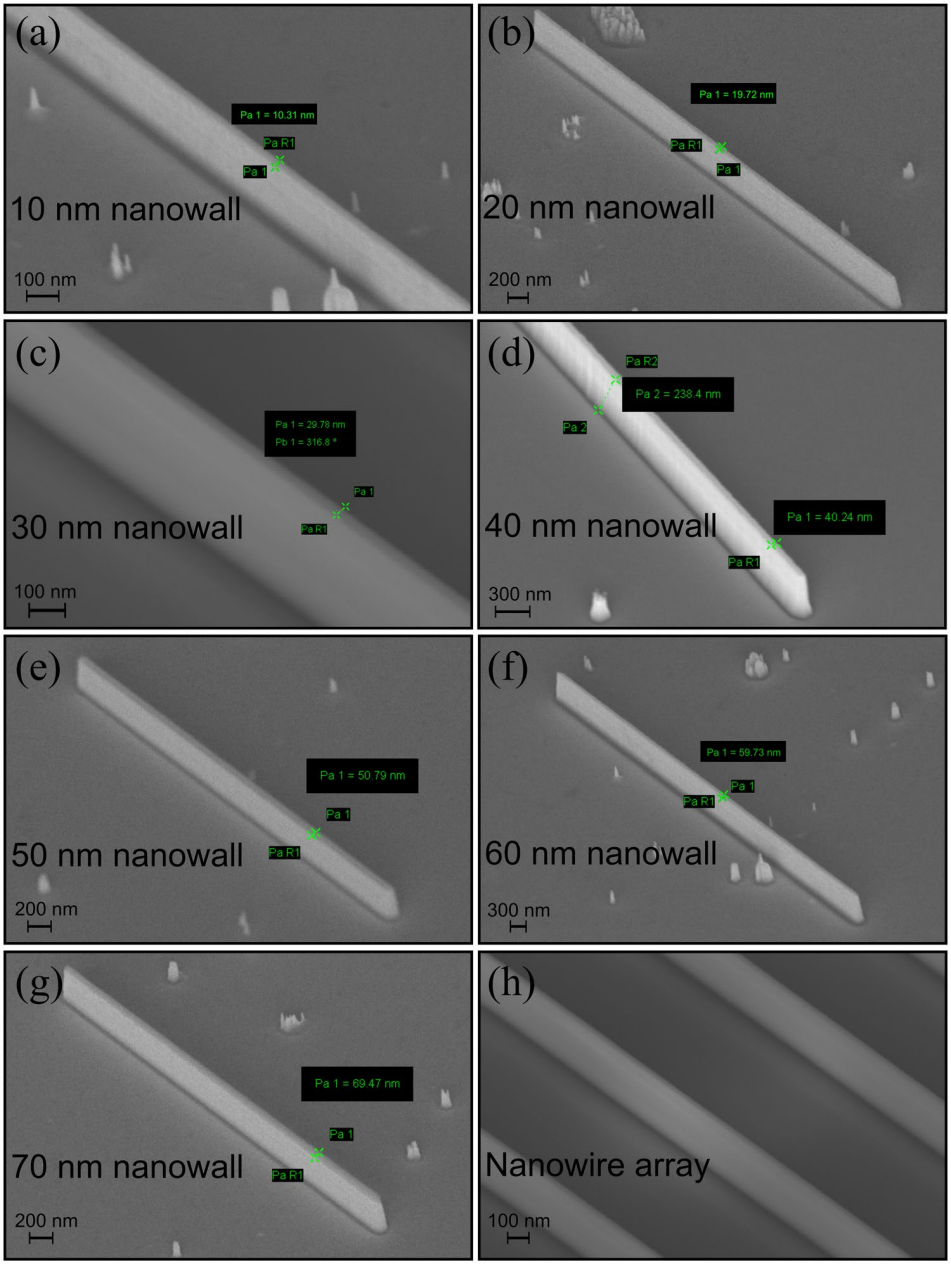
The scanning electron micrographs of fabricated nanowalls of widths (**a**) 10 nm, (**b**) 20 nm, (**c**) 30 nm, (**d**) 40 nm, (**e**) 50 nm, (**f**) 60 nm, and (**g**) 70 nm; (**h**) an SEM image of a typical array of nanowalls, which are used for the PL measurements.

**Figure 4:**
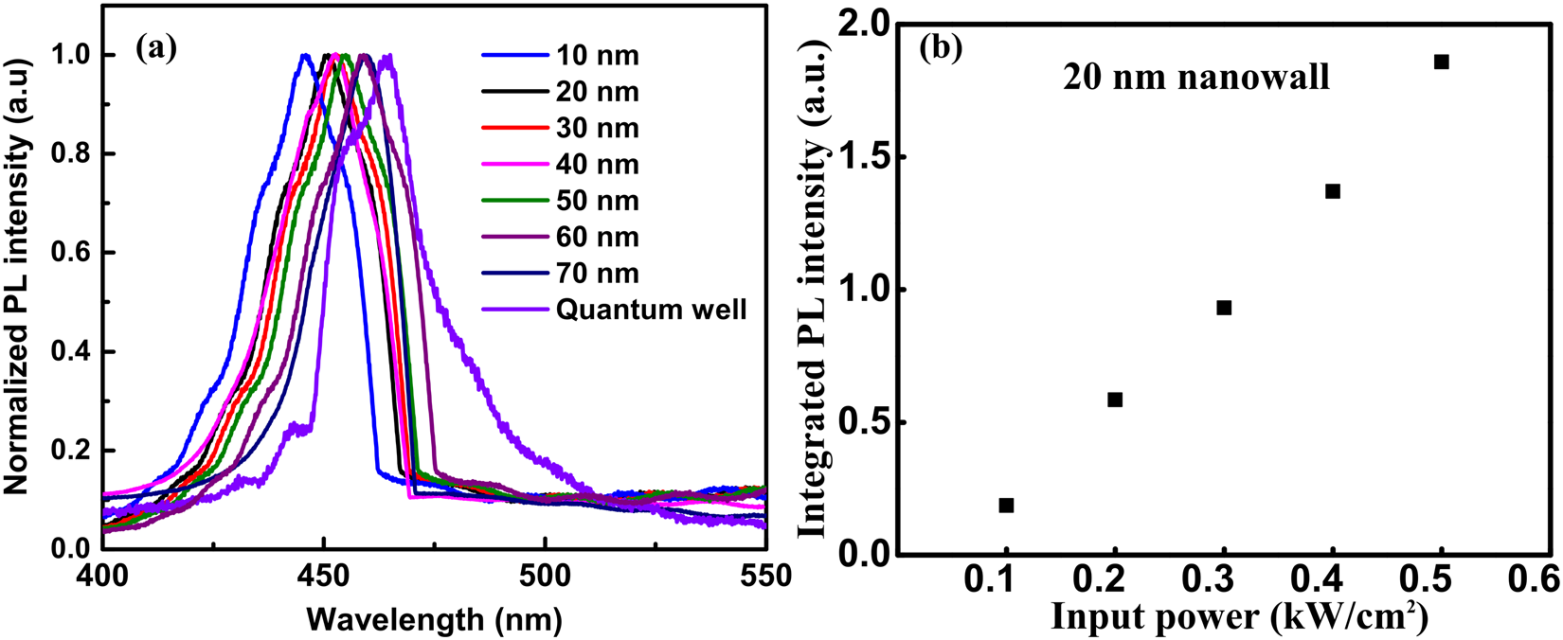
(**a**) PL intensity as a function of wavelength is shown for all the nanowalls; (**b**) integrated PL intensity as a function of laser power is shown for the 20 nm nanowall. The linear dependence of the intensity on input power confirms that the excitonic nature of the emission.

**Figure 5:**
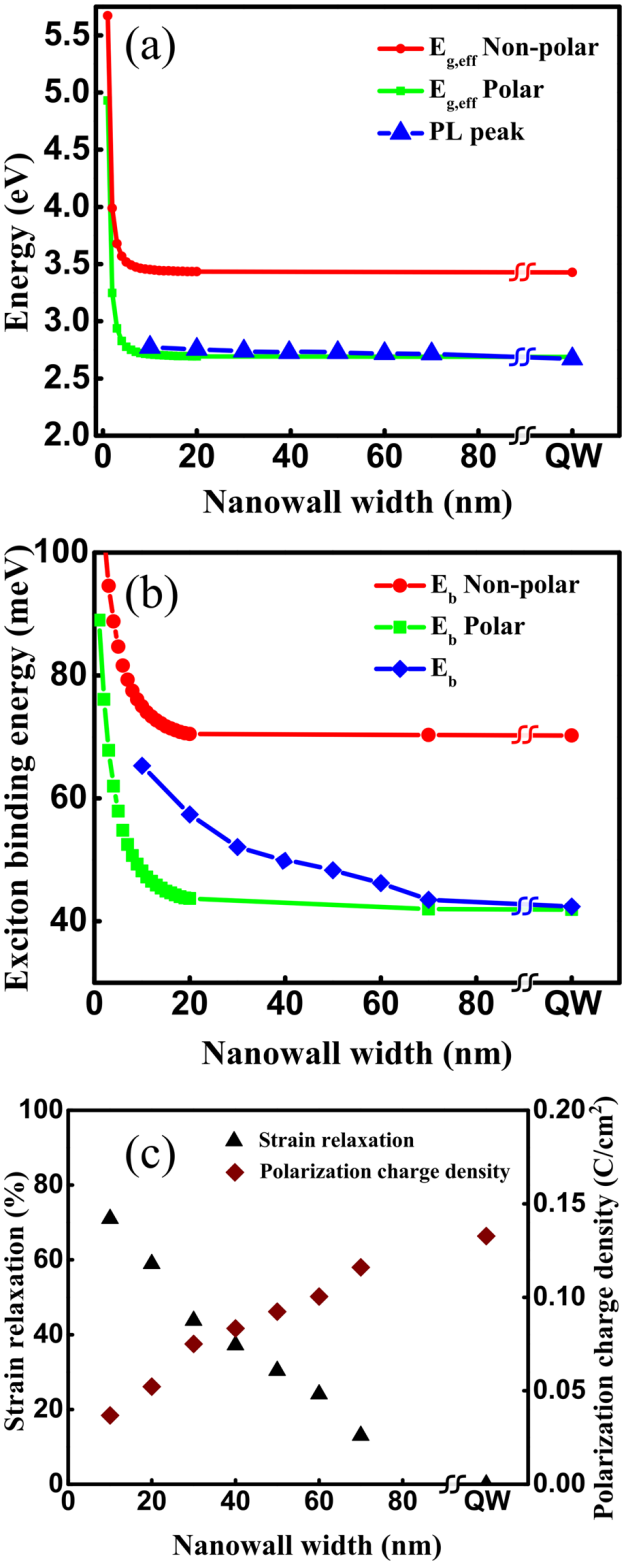
(**a**) The effective energy bandgap for strained and relaxed systems along with the PL peak energies as a function of nanowall width; (**b**) experimentally determined exciton binding energies of the fabricated nanowalls along with theoretically estimated exciton binding energies for strained (polar) and relaxed (nonpolar) systems are plotted as a function of nanowall widths; (**c**) the polarization charge density and corresponding strain relaxation for different nanowall dimensions.

A combination of dry (Ar and Cl_2_) and wet chemical etching (boiling H_3_PO_4_) processes is used to fabricate the nanowalls^28,31–34^. The detailed method for the fabrication of the nanowalls is described in ref.^28^. The nanowalls are finally annealed in N_2_ environment. Nanowalls of 10 to 70 nm widths are fabricated by this method. Figure 3(a–g) show scanning electron microscope (SEM) images of individual nanowalls. The measurements are done on an array (5 × 10) of nanowalls of the same dimension. A typical SEM image of a nanowall array is shown in Fig. 3(h). We have not observed any noticeable change in the nanowall dimension within the same array. Photoluminescence measurements are performed on the fabricated nanowalls. The excitation source used in the experiment is a He-Cd laser with emission wavelength centered at 325 nm and output power of 18 mW for a ~70 µm spot size. A lens tube preceded by a long pass filter is used to couple the signal from the sample to the optical fiber cable, which feeds it to the monochromator and CCD assembly. The signal is digitally processed with the help of an integrated software. For power dependent measurements, a neutral density filter is kept in the path of the laser source, which varied the power levels upon rotation. Figure 4(a) shows the PL spectra for different nanowalls. It can be seen from Fig. 4(a) that the peak wavelength of a 70 nm nanowall is almost the same as that of a quantum well. The position of the peak varies rather slowly with the nanowall width. This can be attributed to a lower Indium composition (10%). Hence, an analytical model that does not consider the in-plane non-uniform distribution of strain can be applied as a first order approximation where the effect is taken into account by considering an average strain along the nanowall width. Nanowalls with higher In composition may require the estimation of position dependent exciton binding energy to determine the effective exciton binding energy for the whole system. The excitonic peak is confirmed through power dependent PL where integrated intensity is found to vary linearly with power. The integrated intensity as a function of power for 20 nm nanowalls in shown in Fig. 4(b). It may be noted that the integrated intensity plot shows an offset, in spite of maintaining linearity at high power, which is due to the poor signal strength at low incident power. Figure 5(a) plots the effective energy bandgap for polar and non-polar systems along with PL peak energies as a function of nanowall width. The theoretical model is used to determine the polarization as a fraction of the fully polarized heterostructure to match the experimental data for the excitonic transitions. It may be noted that matching provides a unique value for a particular dimension of the nanowall. The excitonic peak energies and exciton binding energies are plotted as a function of nanowall width in Fig. 5(b). It may be noted that for wider nanowall the bound state is closer to the effective band-edge with InGaN/InGaN/GaN providing the vertical confinement, which evolves to GaN/InGaN/GaN for smaller dimensions. This is a manifestation of the strain relaxation for smaller width nanowalls.

The piezoelectric polarization ($\delta P$) is related to the basal strain for a c-axis growth as,

$\delta P={e}_{33}{{\epsilon }}_{3}+{e}_{31}({{\epsilon }}_{1}+{{\epsilon }}_{2})$, where ${{\epsilon }}_{3}=(c-{c}_{0})/{c}_{0}$ is the strain along the c axis, in-plane strain ${{\epsilon }}_{1}={{\epsilon }}_{2}=(a-{a}_{0})/{a}_{0}\,$is assumed to be isotropic, *a*_0_ and *c*_0_ are the equilibrium values for the lattice constants, and *e*_33_ and *e*_31_ are the piezoelectric coefficients. Figure 5(c) shows the polarization charge density and corresponding strain relaxation as a function of the nanowall width assuming standard parameters for GaN crystal^18^. The results clearly show the increase in the strain relaxation as the wall width is decreased as intuitively predicted earlier. It may be noted that the strain relaxation can be non-uniform along the width of the nanowall. The current methodology provides an average over the entire dimension, which can be used as a powerful technique for better device design. This method will help us to calculate the average manifested strain relaxation. Our method provides a first order understanding of the effect of strain relaxation, thus serving as a powerful and handy technique for better device design at the proposed dimensions and understanding experimental observations. The strain relaxation is found to be 70% for the 10 nm nanowall.

We have demonstrated strain relaxation and its associated impacts in InGaN/GaN lateral nanowall systems. The exciton binding energy bears the strong signature of the strain relaxation, which can be determined experimentally. The strain relaxation leads to a change in the polarization, which further changes the effective band-edge and excitonic transitions. Schrodinger equation in fractional dimension is used to accurately estimate the exciton binding energy, which helps to correlate exciton binding energy with the strain relaxation. The model takes into account the effect of asymmetric vertical confinement and size dependent lateral barrier profile.
